# *Tipula* (*Vestiplex*) *butvilai* sp. nov., a new crane fly (Diptera, Tipulidae) from Yunnan, China

**DOI:** 10.3897/zookeys.869.34166

**Published:** 2019-08-05

**Authors:** Pavel Starkevich, Aidas Saldaitis, Qiu-Lei Men

**Affiliations:** 1 Nature Research Centre, Akademijos 2, LT-08412 Vilnius, Lithuania Nature Research Centre Vilnius Lithuania; 2 School of Life Sciences, Provincial Key Laboratory of the Biodiversity Study and Ecology Conservation in Southwest Anhui Province, Research Center of Aquatic Organism Conservation and Water Ecosystem Restoration in Anhui Province, Anqing Normal University, Anqing, Anhui 246011, China Anqing Normal University Anqing China

**Keywords:** hypopygium, nematoceran, ovipositor, taxonomy, Tipuloidea

## Abstract

A new crane fly, Tipula (Vestiplex) butvilai**sp. nov.**, is described and illustrated based on new material collected in the Nu Jiang Valley of Yunnan Province, China. The hypopygium for the most closely related species, Tipula (Vestiplex) testata Alexander, 1935, is also illustrated. A key is provided to distinguish males of the new species from those of other species in the T. (V.) bicornigera Alexander, 1938 species group.

## Introduction

The world fauna of the subgenus Tipula (Vestiplex) Bezzi, 1924 including the type species, *Tipula cisalpina* Riedel, 1913, currently contains 156 described species distributed throughout the Nearctic, Palaearctic and Oriental Regions (Oosterbroek 2019). The Chinese fauna of T. (Vestiplex) is richly represented, with 69 species and one subspecies (Oosterbroek 2019).

Females belonging to the subgenus T. (Vestiplex), are characterized by having an ovipositor with a powerfully constructed and heavily sclerotised cerci and serrate outer margins, although margins may be smooth in several Asiatic species. The hypovalvae are small to rudimentary (Alexander 1935, 1965, Alexander and Byers 1981). The male genitalia are extremely polymorphic (Savchenko 1964), typically with tergite 9 forming a shallowly concave and sclerotised saucer, although some other species have their tergite 9 completely divided longitudinally by a pale membrane (Alexander 1935, Alexander and Byers 1981).

Tipula (Vestiplex) can be separated into various species groups based on hypopygium structures. The group of species with a saucer-shaped tergite 9 has been investigated by several authors (Mannheims 1953; Hemmingsen 1956; Savchenko 1960, 1964; Theowald and Mannheims 1963;), with seven species groups currently recognized: T. (V.) coquillettiana, T. (V.) erectiloba, T. (V.) excisa, T. (V.) leucoprocta, T. (V.) nubeculosa, T. (V.) scripta, and T. (V.) virgatula. Taxonomic studies of species with a longitudinally divided tergite 9 are still unresolved. Edwards (1928) proposed the T. (V.) himalayensis species subgroup of the T. (V.) arctica group for T. (V.) avicularia Edwards, 1928. Alexander later adopted T. (V.) himalayensis as a species group and included a number of species with a divided tergite 9 (Alexander, 1932, 1933, 1934, 1935, 1936, 1959, 1963). Savchenko (1960) also proposed several species groups: T. (V.) avicularia, T. (V.) divisotergata, and T. (V.) subtincta, for various Asiatic species based on features of the male hypopygium.

A revisionary study of this subgenus on a global basis was conducted by Starkevich (2012) and a phylogenetic review of the group is ongoing. Those species with a divided tergite 9 were grouped into the following species groups according to the phylogenetic tree: T. (V.) hymalayensis, T. (V.) avicularia, T. (V.) divisotergata, T. (V.) subtincta, T. (V.) eurydice and T. (V.) deserrata. Some species placements remain unresolved due to a lack of fresh material. As part of an ongoing morphological analysis, the Tipula (Vestiplex) bicornigera Alexander, 1938 species group is proposed and diagnosed herein for the first time. The new species, belonging to this group, was detected while sorting and identifying specimens of T. (Vestiplex) from China’s Sichuan and Yunnan Provinces.

## Materials and methods

Adult crane flies were collected at night using an ultraviolet light trap and preserved in 96% ethanol. Specimens were studied with a Nikon SMZ800 stereomicroscope. Pictures were taken with an INFINITY–1 camera mounted on a Nikon Eclipse 200 stereomicroscope and Canon EOS 80D mounted on an Olympus SZX10 dissecting microscope. Genitalia were studied after boiling them in 10% NaOH solution for 5–10 minutes.

Descriptive terminology generally follows that of Alexander and Byers (1981) and Frommer (1963) with some additions for particular features for some T. (Vestiplex). The term appendage of sternite 9 (A9S) is adopted from Mannheims (1953), and terms ventral lobe and dorsal lobe of A9S were adopted from Gelhaus (2005).

Abbreviations for institutional collections used herein: **USNM**United States National Museum of Natural History, Washington, DC, USA; **NRC** Nature Research Centre, Vilnius, Lithuania; other abbreviation: **PS** slide Pavel Starkevich.

## Taxonomy

### Tipula (Vestiplex) bicornigera species group

The *bicornigera* group can be easily distinguished from other T. (Vestiplex) species by a remarkable tergite 9 with its ventral part flattened and hypertrophic (Figs 5, 16). The following species, all distributed in China (Sichuan, Hubei and Taiwan), are placed in the T. (V.) bicornigera group: Tipula (Vestiplex) bicornigera Alexander, 1938, Tipula (Vestiplex) subtestata Alexander, 1938, Tipula (Vestiplex) testata Alexander, 1935 and Tipula (Vestiplex) xingshana Yang & Yang, 1997. Males of the *bicornigera* group can be recognized by the following features: tergite 9 divided by a pale membrane into two parts, ventral part shaped as a hypertrophic and flattened plate, its inner and posterior margin blackened, and microscopically roughened; each half of dorsal part of tergite 9 with a posterior lobe on the posterior margin or with an additional posteromedial lobe which is covered with setae and pointed caudad; gonocoxite dorsally produced into a black spine; sternite 9 with A9S dorsal lobe usually reduced into a small triangular or rod-shaped sclerite , or narrow and distinct in case of T. (V.) butvilai sp. nov.

### Key to species (male) of the *bicornigera* group

**Table:** 

1	Each half of dorsal part of tergite 9 with a single posterior lobe on posterior margin (Figs 2, 14)	**2**
–	Each half of dorsal part of tergite 9 with two lobes on posterior margin (Alexander 1938a: pl. 2, fig. 26; Alexander 1938b: pl. 2, fig. 30; Yang and Yang 1997: fig. 3a)	**3**
2	Mesonotal prescutum golden yellow with three olive-brown stripes that are poorly defined against the background; femur without preapical yellow ring; inner gonostylus with small lower beak and dorsal crest rounded (Fig. 19); dorsal lobe of A9S in the shape of a short process (Fig. 20)	**Tipula (Vestiplex) testata Alexander, 1935**
–	Mesonotal prescutum yellowish with four olive-yellow stripes that are narrowly bordered by yellow; femur with yellow preapical ring (Fig. 1); inner gonostylus without lower beak; dorso-median margin with blackened tooth; dorsolateral margin in the shape of blackened obtuse outgrowth; dorsal crest nearly rectangular (Fig. 8); dorsal lobe of A9S long and narrow (Fig. 4)	**Tipula (Vestiplex) butvilai sp. nov.**
3	Antenna with first flagellar segment brown; femur with preapical yellow ring	**Tipula (Vestiplex) bicornigera Alexander, 1938**
–	Antenna with basal half of first flagellar segment yellow; femur without preapical yellow ring	**4**
4	Mesonotal prescutum with four reddish brown stripes	**Tipula (Vestiplex) subtestata Alexander, 1938**
–	Mesonotal prescutum with three pale grayish stripes	**Tipula (Vestiplex) xingshana Yang & Yang, 1997**


#### Tipula (Vestiplex) butvilai

Taxon classificationAnimaliaDipteraTipulidae

Starkevich, Saldaitis & Men sp. nov.

82616b84-215d-56ad-bdd6-96045dd7dfdd

http://zoobank.org/FA8969F2-485F-4FCD-AFF4-D11CD9C977D2

Figs 1 , 2–8 , 9–13

##### Holotype.

male, China, NW. Yunnan, Nu Jiang Valley, S. from Gongshan, elevation 2100 m, 27°43.42'N, 98°45.15'E, 15–16.v.2018, leg. Butvila & Saldaitis (NRC).

**Figure 1.**
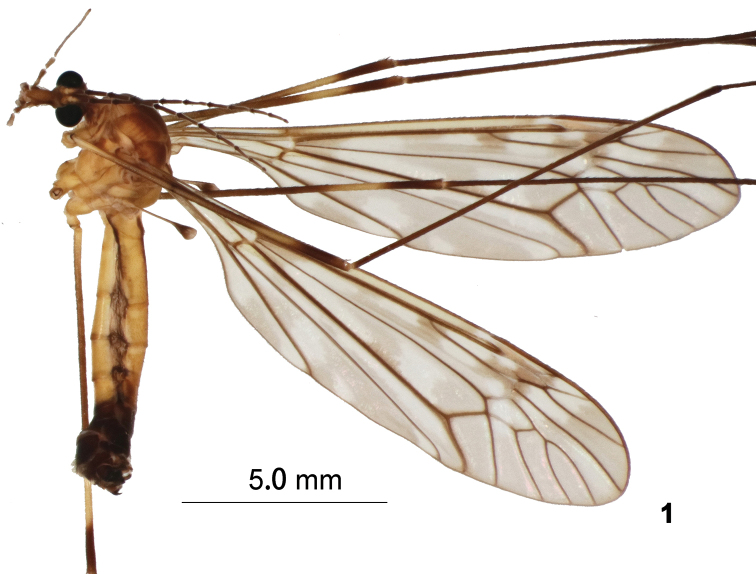
T. (Vestiplex) butvilai sp. nov., adult male, lateral view of holotype.

##### Paratypes.

1 male and 3 females topotypic, male genitalia slide No. PS0419m, female genitalia slide No. PS0420f (NRC), same data as holotype.

##### Diagnosis.

Among other members of T. (V.) bicornigera group T. (V.) butvilai sp. nov. can be recognized by yellow body, elongate antennae which if bent backward reach the base of the abdomen, brown flagellum and femur with a preapical yellow ring. Dorsal portion of tergite 9 posteriorly with a pair of oval lobes. Appendage of sternite 9 with dorsal lobe blackened, long and narrow. Sternite 8 provided with long setae.

##### Description.

Adult male (Fig. 1) (*N* = 2). General body coloration yellow. Body length 11.9–12.4 mm, wing length 14.1–14.9 mm.

*Head*. Yellowish, vertex and occiput with dark brown median line (Fig. 1). Rostrum yellow, short, nasus inconspicuous. Antenna 13-segmented, elongate, if bent backward reaching base of abdomen; scape, pedicel yellow, first flagellomere basally yellow, the rest of flagellum brown. Flagellar segments except first one with basal enlargements. Verticils slightly longer than corresponding segments. Palpus yellowish.

*Thorax*. Pronotum yellow. Mesonotal prescutum, yellowish, with 4 olive-yellow longitudinal stripes, narrowly bordered by yellow and median pair separated by brownish interspace. Scutum yellow, scutal lobes each with two olive-yellow spots bordered by yellow. Scutellum and mediotergite yellow with dark median line. Pleura yellow. Leg with coxa and trochanter yellow; femur brown with broad preapical yellow ring, tip dark brown; tibia and tarsal segments dark brown; tarsal claw with tooth. Wing (Fig. 1) yellowish-brown, cells c and sc slightly darker than ground color; stigma brown; Rs suffused with dark brown at origin point and the level of its branch; discal cell transparent with the exception of outer end which suffused with brown; apical half of cells r_3_, r_4_ and r_5_, and entire cells m also suffused with brown, some large hyaline areas at cells cup and a_1_. Venation: R_1+2_ entire, discal cell narrow, elongated, petiole of cell m1 distinctly shorter than discal cell. Halter pale yellow with brown knob.

*Abdomen*. Abdominal segments 1–5 yellow, with dorsal and lateral lines, remaining segments brownish black. Tergites 6–7 laterally pale, sternites 6–7 with posterior margin pale.

**Figures 2–8.**
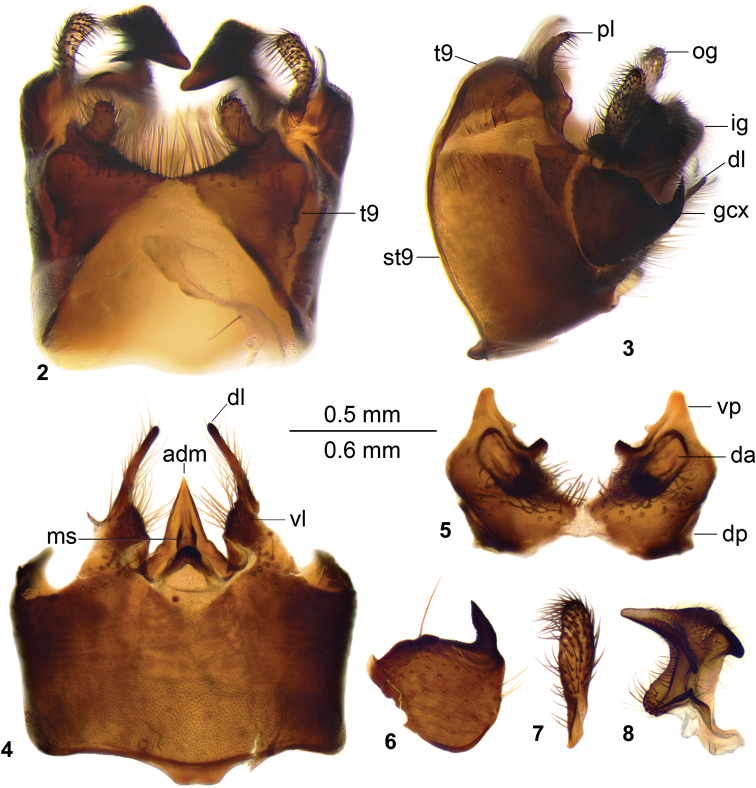
Hypopygium of male T. (Vestiplex) butvilai sp. nov. **2** hypopygium, dorsal view **3** hypopygium, lateral view **4** sternite 9, ventral view (tergite 9, gonocoxites, left outer and inner gonostyles removed) **5** tergite 9, dorsal view **6** left gonocoxite **7** left outer gonostylus **8** left inner gonostylus, lateral view. Abbreviations: adm, adminiculum; da, depressed area; dl, dorsal lobe of appendage of sternite 9; dp, dorsal portion of tergite 9; gcx, gonocoxite; ig, inner gonostylus; ms, median sclerite; og, outer gonostylus; pl, posterior lobe of dorsal portion of tergite 9; st9, sternite 9; t9, tergite 9; vl, ventral lobe of appendage of sternite 9; vp, ventral portion of tergite 9. Scale bar: 0.6 mm (**2–3**), 0.5 mm (**4–8**).

*Hypopygium.* Brownish black. Tergite 9 completely divided at midline by pale membrane (Figs 2, 6). Posterior margin with V-shaped notch; posterolateral margin of tergite 9 triangular; dorsal portion with posterior margin of tergite 9 covered with setae, with a pair of lobes oval in dorsal view directed caudad (Figs 2, 3). Ventral portion of tergite 9 hypertrophied, occupying ~half of entire tergite area. Small blackened glabrous process on either side of midline, a depressed oval area on each half of ventral portion of tergite 9. Gonocoxite entirely separate from sternite 9, dorsally produced into curved spine with acute tip (Figs 3, 6). Outer gonostylus club-shaped (Fig. 7). Inner gonostylus blackened, lower beak missing, distal surface covered with setae; upper beak straight triangular, dorsal crest nearly rectangular in ventral view; dorso-median margin proximally from upper beak produced into blackened tooth; dorsolateral margin bent outwardly forming blackened obtuse outgrowth (Fig. 8). Appendage of sternite 9 provided with setae, with dorsal lobe blackened, long and narrow, ventral lobe nearly triangular (Fig. 4). Adminiculum triangular in ventral view, median sclerite) anteriorly with elevated margin (Fig. 4). Strenite 8 provided with long setae.

##### Female.

Body length 18.1–19.2 mm, wing length 16.6–18.1 mm (*N* = 3). Generally similar to male. Antenna short, if bent backward reaching pronotum. Scape and pedicel yellow, flagellum brown, flagellar segments cylindrical. Abdomen yellow, dorsal abdominal stripe broad, black.

**Figures 9–13.**
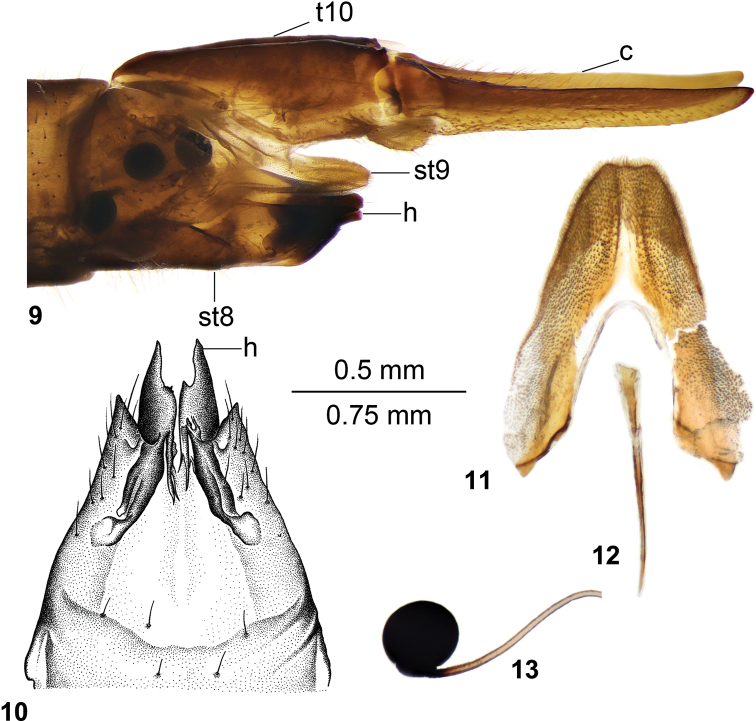
Ovipositor of female T. (Vestiplex) butvilai sp. nov. **9** ovipositor, left lateral view **10** sternite 8 with hypovalvae, ventral view **11** sternite 9, dorsal view **12** furca, dorsal view **13** spermatheca, lateral view. Abbreviations: c, cerci; h, hypovalvae; st8, sternite 8; st9, sternite 9; t10, tergite 10. Scale bar: 0.75 mm (**9**), 0.5 mm (**10–13**).

*Ovipositor* (Figs 9–13). Tergite 10 light brown. Cercus yellow, nearly straight, with tip acute and outer margin with rough indistinct serration (Fig. 9). Sternite 8 brown, pale area before hypovalvae (Fig. 10). Hypovalva strongly sclerotised, black, shaped posteriorly as an obliquely truncated plate with acute tip, anteriorly nearly rectangular, with additional submedian denticle on inner side in ventral view. Lateral angle of sternite 8 strongly sclerotised, nearly triangular and acute, covered with setae. Median incision between hypovalvae with serrated medial area. Sternite 9 with lateral sclerites nearly straight, obtuse anteriorly, the surface is covered by short setae (Fig. 11). Furca long and narrow (Fig. 12). Three spherical spermathecae (Fig. 13).

##### Comparative material examined.

Tipula (Vestiplex) bicornigera Alexander, 1938: holotype, male, China, Taiwan, Oiwake, Noko-gun, altitude 7570 feet [2307 m], August 12, 1936 (Takahashi) (USNM); Tipula (Vestiplex) subtestata Alexander, 1938: holotype, male, China, Sichuan, Mount Omei, Chu Lao Tong Temple, altitude 6500 feet [1981 m], June 5–6, 1937 (Tsen); paratype, the same data as holotype (USNM); Tipula (Vestiplex) testata Alexander, 1935: holotype, male, China, Beh-Luh-Din, 30 miles north of Chengdu, altitude 6000 feet [1829 m], August 8–10, 1933 (Graham); paratypes, 3 males, topotypic, August 12–17, 1933 (Graham) (USNM).

##### Biology and distribution.

Two males and three females were collected during mid May, 2018. All were collected at ultraviolet lights over two nights in a Nu Jiang (Salween) river valley in the northwestern part of China’s Yunnan Province bordering North Myanmar (Kachin State). The new species was collected at altitudes of approximately 2100 meters in mixed mountain forest, dominated by various deciduous trees, bamboo and bushes (Figs 21–22).

##### Discussion.

Tipula (V.) butvilai sp. nov., is closest to T. (V.) testata (China, Sichuan) based on the shape of the male hypopygium. Both species are characterized by their hypertrophied ventral portion of tergite 9 and a pair of lobes on posterior margin of dorsal portion of tergite 9 but can be easily separated by the shape of the appendage of sternite 9, inner gonostylus and details of tergite 9. They can also be separated by the yellow preapical ring on the femur which is present in T. (V.) butvilai sp. nov., but absent in T. (V.) testata. Other species closely related to T. (V.) butvilai sp. nov., and T. (V.) testata are T. (V.) bicornigera (Taiwan; Alexander 1938a: pl. 2, fig. 26), T. (V.) subtestata (China, Sichuan; Alexander 1938b: pl. 2, fig. 30) and Tipula (Vestiplex) xingshana Yang & Yang, 1997(China, Hubei; Yang and Yang 1997, p. 1438, fig. 3a–c) which all share a hypertrophied ventral portion of tergite 9, but can be separated by the shape of posterior margin of the dorsal portion of tergite 9, which includes two pairs of lobes.

##### Etymology.

The new species is named after our colleague and prominent Lithuanian collector Rimantas Butvila (Joniškis, Lithuania).

#### Tipula (Vestiplex) testata

Taxon classificationAnimaliaDipteraTipulidae

Alexander, 1935

3ac12b40-45a3-5058-afab-16ee4109c136

Figs 14–20

Tipula (Vestiplex) testata Alexander, 1935: 119; Tipula (Vestiplex) testata: Savchenko 1964: 157; Tipula (Vestiplex) testata: Oosterbroek and Theowald 1992: 159.

##### Material examined.

1 male, China, W. Sichuan, road Yaan/Kangding, Erlang Shan Mt., 29°52.23'N, 102°18.35'E, elevation 2100 m, 10–11.IV.2010, genitalia slide No. PS0421m, leg. A. Saldaitis; 5 males, W. Sichuan, road Yaan/Kangding, Erlang Shan Mt., 30°32.40'N, 102°46.10'E, elevation 2161 m, 20.VIII.2014, leg. Floriani & Saldaitis (NRC).

**Figures 14–20.**
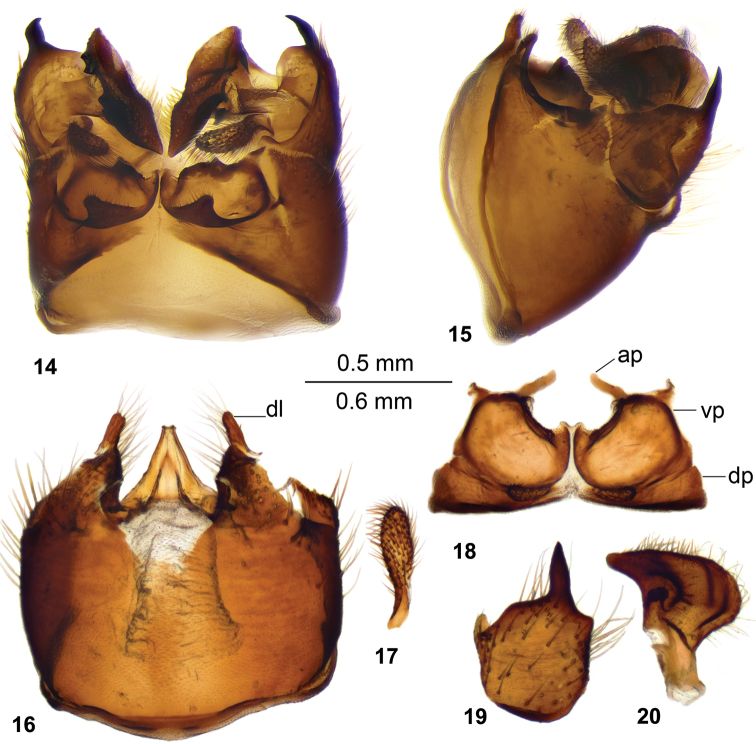
Hypopygium of male T. (Vestiplex) testata. **14** Hypopygium, dorsal view **15** hypopygium, lateral view **16** sternite 9, ventral view (tergite 9, gonocoxites, outer and inner gonostylus removed) **17** left outer gonostylus **18** tergite 9, dorsal view **19** left gonocoxite **20** left inner gonostylus, lateral view. Abbreviations: ap, anal plates; dl, dorsal lobe of appendage of sternite 9; dp, dorsal portion of tergite 9; vp, ventral portion of tergite 9. Scale bar: 0.6 mm (**14–15**), 0.5 mm (**16–20**).

*Hypopygium.* Black. Tergite 9 completely divided at midline by pale membrane (Figs 14, 16). Posterior margin with U-shaped notch, posterolateral margin of tergite 9 rounded. Anal plate a short process (Fig. 16). Dorsal portion with posterior margin of tergite 9 with a pair of lobes directed caudad and covered with setae. Ventral portion of tergite 9 flattened, hypertrophied and occupying almost entire tergite area. Each half of ventral portion of tergite 9 shallowly concaved and with blackened rim. A blackened glabrous area on either side of midline dividing tergite 9. The inner margin of midline slightly elevated, terminated into short obtuse process. Gonocoxite entirely separate from sternite 9, dorsally produced into gently curved spine with acute tip (Figs 15, 17). Outer gonostylus club-shaped (Fig. 18). Inner gonostylus brown, lower beak small, upper beak short, dorsal crest rounded (Fig. 19). Appendage of sternite 9 covered with setae, with short dorsal lobe, ventral lobe nearly triangular (Fig. 20). Adminiculum triangular in ventral view with median sclerite (Fig. 20).

**Figures 21, 22.**
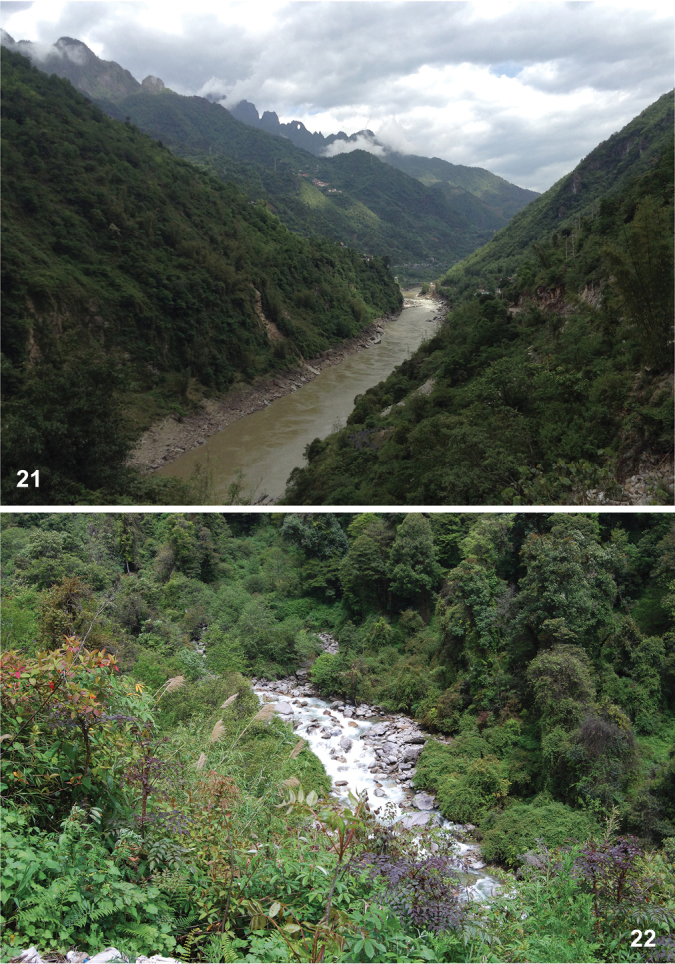
Type locality of T. (Vestiplex) butvilai sp. nov., China, NW. Yunnan, Nu Jiang valley, S. from Gongshan, 27°43.42'N, 98°45.15'E.

## Supplementary Material

XML Treatment for Tipula (Vestiplex) butvilai

XML Treatment for Tipula (Vestiplex) testata
